# Rapid identification, capsular typing and molecular characterization of *Streptococcus pneumoniae* by using whole genome nanopore sequencing

**DOI:** 10.1186/s12866-020-02032-x

**Published:** 2020-11-13

**Authors:** S. Garcia-Garcia, A. Perez-Arguello, D. Henares, N. Timoneda, C. Muñoz-Almagro

**Affiliations:** 1 Institut de Recerca Sant Joan de Deu, Hospital Sant Joan de Deu, Barcelona, Spain; 2 CIBER de Epidemiologia y Salud Publica (CIBERESP), Instituto de Salud Carlos III, Madrid, Spain; 3 Departament de Medicina, Universitat Internacional de Catalunya, Barcelona, Spain

**Keywords:** Whole genome sequencing, MinION, Capsular typing, Wg-MLST, *Streptococcus pneumoniae*

## Abstract

**Background:**

Whole genome sequencing has emerged as a useful tool for identification and molecular characterization of pathogens. MinION (Oxford Nanopore) is a real-time third generation sequencer whose portability, affordability and speed in data production make of it an attractive device for whole genome sequencing. The objective of this study is to evaluate MinION sequencer for pathogen identification and molecular characterization of *Streptococcus pneumoniae* isolated at a children’s Hospital. Whole genome sequencing of 32 *Streptococcus pneumoniae* invasive isolates, previously characterized by standard methods (Quellung reaction, Multiplex PCR and Sanger-MLST), were performed. DNA was extracted using ZymoBIOMICS DNA Microprep kit. Quantification and purity of DNA was assessed by Qubit and Nanodrop, respectively. Library preparation was performed using the Rapid Barcoding Kit. Real-time workflow EPI2ME platform “What’s it in my pot” was used for species identification. Fast5 sequences were converted into FASTQ by Albacore software. Reads were assembled using CANU software. PathogenWatch, genomic epidemiology and pubmlst online tools were used for capsular typing and/or whole genome-MLST profile.

**Results:**

Rapid identification of *Streptococcus pneumoniae* was achieved by “What’s in my pot”. Capsular typing was correctly assigned with PathogenWatch in all 32 isolates at serogroup level and 24 at serotype level. Whole genome-MLST results obtained by genomic epidemiology and pubmlst were consistent with double locus variant clonal complex obtained by Sanger-MLST in 31 isolates.

**Conclusion:**

MinION sequencer provides a rapid, cost-effective and promising pathway for performing WGS by a pocked-sized device for epidemiological purposes but improving its sequencing accuracy will make it more appealing to be used in clinical microbiology laboratories.

**Supplementary Information:**

The online version contains supplementary material available at 10.1186/s12866-020-02032-x.

## Background

*Streptococcus pneumoniae* is responsible for causing disease especially in children younger than 5 years and the elderly. It is associated with high morbidity and mortality worldwide [1]. Updated regional mortality estimates were published by WHO for 2008, estimating 541,000 pneumococcal deaths in children that year [2]. *S.pneumoniae* is a highly adaptable microorganism evolving continuously. The replacement phenomenon against vaccine strategies, referring to the expansion of non-vaccine serotypes as a result of the removal from the population of vaccine types, should be surveillance with a rapid and accurate diagnosis and molecular characterization. Traditional methods for molecular epidemiology have several limitations including a possible untimely delivery of results [3]. A conventional *S.pneumoniae* typing system is based on multi locus sequence typing (MLST). MLST is a procedure for characterizing isolates using the partial sequence of seven well-conserved housekeeping genes by Sanger technology. A sequence type (ST) is assigned by comparing each gene against other isolate profiles in the public MLST reference database (https://pubmlst.org/). STs are grouped into clonal complexes by their similarity to a central allelic profile [4]. Using conventional Sanger sequencing approaches, MLST turn out a laborious technique, expensive and difficult to perform at scale.

To date, at least 100 pneumococcal polysaccharide capsule types have been described [5]. The ability of pneumococci to cause disease is directly related to the polysaccharide capsule, the major virulence factor. Pneumococci can be classified into different serotypes according to their capsule type using conventional serotyping techniques. On the one hand multiplex PCR combined with both fragment analysis and automated fluorescent capillary electrophoresis. On the other hand, the gold standard Quellung reaction to complete serotyping [6].

Next generation sequencing technology provides lots of information about species, serovar, virulence, pathogenicity, antimicrobial resistance in just one sequencing run [7]. Whole genome sequencing (WGS) is evolving as a routine practice as a diagnostic. The affordable cost and the reduced turnaround time in comparison to standard methods are achieving a viable and promising technology in diagnostic. NGS is characterized by higher throughput, increased read lengths, reductions in sequencing time and low overall cost when compared to conventional DNA sequencing methods [8]. The main extra feature of third generation sequencing, which includes MinION sequencer, is the long-read length. In 2014, Oxford Nanopore Technologies launched MinION, the only pocket-sized portable real-time device for DNA and RNA sequencing. It is the smallest sequencing device available. It plugs directly into a PC or laptop via USB cable. Low hardware requirements mean a standard computer is sufficient for sequencing. Nanopore-based sequencing relies on changes in the ionic current of nucleotides as a single DNA molecule passes through artificial nanopores as a single DNA molecule [9] [10]. Nevertheless, the high error rate exhibited by MinION sequencer has limited its ability to compete with existing sequencing technologies but software developments are continuously increasing base call accuracy [11].

The work described in this study is meant to provide information on the state of MinION sequencer for assessing its capabilities for pathogen identification and molecular characterization of *S.pneumoniae* isolated at a children’s Hospital.

## Results

### Comparison between Nanopore technology and conventional methods

A rapid and correct identification of all 32 *S.pneumoniae* invasive strains was achieved by WIMP workflow in only 5 min, meanwhile at least two days are required by traditional bacterial identification. WGS took around 3 h. Table 1 includes the length of time taken for each step comparing Nanopore technology and conventional microbiology approach.

**Table 1:** MinION and Sanger workflow times comparison

Steps	MinION workflow	Sanger workflow
Sample preparation	Strains cultured overnight and harvested the following day	
DNA extraction	2 h	40 min
Preparation for sequencing	1 h	8h15min
Sequencing	5 min-3 h*	6 h
Data analysis	1 h	2 h
Total	4 h5 min-7 h	16 h45min

Clarifications for Sanger workflow: a) “Preparation for sequencing” step includes four different steps which make it more laborious [amplification (3h30min), 1st purification (1 h15min), sequence reaction (3 h) and 2nd purification (30 min)]; b) “Sequencing” step only includes the seven housekeeping genes: aroE, gdh, gki, recP, spi, xpt and ddl

^*^Five minutes are enough for identification at species level and at least 3 h are required to obtain whole genome data. However, we always initiated a standard 18 h sequencing procedure

*Abbreviations*: *NA* not available, *WGS* whole-genome sequencing

Regarding prices of both workflows, the cost per sample characterized by MinION is about 65.11€, meanwhile by Sanger is roughly 53.40€.

As depicted in Table 2, when comparing MLST derived from Sanger or Nanopore data, a concordance of 16% was achieved in those strains that shared seven out of seven genes (ST). The concordance at SLV and DLV level was 56 and 25%, respectively. A match of four out of seven genes (QLV) was solely obtained in one strain (3%). Wg-MLST results obtained by genomic epidemiology and pubmlst online tools were consistent with at least DLV definition obtained by Sanger-MLST in 31/32 *S.pneumoniae* invasive strains. Capsular typing was correctly assigned with PathogenWatch in all 32 isolates at serogroup level and 24 at serotype level. Limitations in the resolution of genotypic inference of serotype are depicted in Table 3.

**Table 2:** *S. pneumoniae* clonal characterization by MinION

Sequence type or clonal complex designation	% of ***S. pneumoniae*** strains corresponding to each designation
ST	16% (5/32)
SLV	56% (18/32)
DLV	25% (8/32)
QLV	3% (1/32)

To obtain the % of *S. pneumoniae* strains corresponding to each designation, MinION results were compared to those obtained by Sanger sequencing. A total of 32 invasive pneumococcal strains were analyzed. Wg-MLST results were achieved in 3 h

*Abbreviations*: *ST* Sequence Type, *SLV* Single Locus Variantm, *DLV*, Double Locus Variant, *QLV* Quadruple Locus Variant

**Table 3:** Serotype comparison between conventional methodology and PathogenWatch online tool

Serotype by conventional methodology	Serotype by MinION (PathogenWatch)
6C	6D
9 V	9A
9 V	9A
9 V	9A
18C	18B
24F	Serogroup 24
24F	Serogroup 24
24F	Serogroup 24

Conventional methodology refers to multiplex PCR combined with fragment analysis and automated fluorescent capillary electrophoresis and Quellung reaction, as explained previously in the methods.

## Discussion

In this study we have carried out an evaluation panel of 32 isolates including pathogen identification and pneumococcal clonal characterization. The whole-genome sequencing turnaround time, from DNA extraction and library preparation to sequencing and data analysis, is roughly 2 days. Nevertheless, it comprehensively identified pathogens at species level in just 5 min. Although there is an increasing trend of microbiologist using MALDI-TOF for bacterial identification in clinical settings, acquiring it requires a high economic investment not affordable for everyone. It is a good and reliable choice but it is not still available in too many laboratories. The sequencing approach using MinION, whereby results are generated and analysed in real time, has the potential to be much faster than Sanger technology. NGS technologies are increasingly being integrated into patient care and clinical management.

Until now Sanger sequencing has been the gold standard only for genotyping not serotyping in clinical laboratories serving as the conventional method whereby NGS data should be compared and validated [12]. WGS is a revolutionary technology that is increasingly replacing traditional clinical diagnostics. It has emerged as a useful tool for identification and molecular characterization of pathogens. However, based on the low concordance in MLST between Sanger and Nanopore data, MinION is not accurate enough to be completely replaced by conventional methods for molecular epidemiology in surveillance programs. Nevertheless, since WGS is becoming increasingly ubiquitous, conventional MLST by Sanger sequencing concept has been extended to include, apart from 7 housekeeping genes, many hundreds or even thousands of loci coined wgMLST [13].

In addition, its capability to produce longer reads and its low price compared to other sequencers make of it an attractive device for WGS. The major advantages are the price, the speed in data production, which would make it appropriate in a hospital for rapid diagnosis, and the ability to sequence a single isolate rather than wait to fill a lane of an Illumina sequencer. Regarding the price, as mentioned elsewhere, due to a single flow cell can be used until twice, the cost per 2 runs (12 samples per run; 24 samples) is around 1562.66€; 65.11€ per sample. Alike, the cost per sample characterized by Sanger workflow is roughly 53.40€. However, several considerations have to be taken into account, namely MinION provides information about the entire pathogen’s genome, meanwhile Sanger characterization is only focus on seven housekeeping genes. Moreover, the time invested in preparation for sequencing is vastly higher in Sanger workflow than that of MinION. The same thing happens with the sequencing time, which is higher 2-fold with Sanger. For these reasons, the total invested time for Sanger workflow is increased 4-fold. Nevertheless, as a limitation of the study, three serogroup 24 were not correctly matched between conventional and MinION methodologies. This event could be explained due to the genome-based method can not subtype serogroup 24. This is not a discrepancy but a limitation in the genome-based serotyping method [14].

Although the approach has great potential, challenges remain. Firstly, the main limitation of MinION sequencing is its lower read accuracy when compared to short-read technologies. While Illumina’s quality PHRED score is about 30, MinION moves around 10 (**Supplementary table**) [8]. Because of that, MinION still has not moved into routine clinical practice. Secondly, strains were collected from our laboratory biobank where they are stored once identified and characterized. Thus, isolates did not proceed directly from clinical samples. Its issue is referring to low-input procedures that notably reduce the DNA requirement for nanopore sequencing. Thirdly, we tested up to 24 strains per flow cell. It might induce to cross contamination between two consecutive runs. However, the price per sequenced strain undoubtedly decreases as a same flow cell is used twice. Finally, with the automation of bioinformatic pipelines, this method could become very attractive for monitoring invasive *S.pneumoniae* strains. The great amount of information generated with WGS can be easily valued with, for example, the analysis of molecular evolution of the isolates, the identification of putative vaccine targets in addition to the detection of antibiotic resistance and virulence genes [15].

## Conclusion

In conclusion, MinION sequencer provides a rapid, cost-effective and promising pathway for performing WGS by a pocked-sized device for epidemiological purposes but improving its sequencing accuracy will make it more appealing to be used in clinical microbiology laboratories.

## Methods

### Study setting and design

The study was conducted at University Hospital Sant Joan de Deu Barcelona (Spain) during 2018. The setting study is a 318-bedsize reference university children’s hospital that attends a paediatric reference population of approximately 300,000 subjects. In 2009, the Molecular Microbiology Laboratory was designated by the government of Catalonia (Spain) as the Catalan support laboratory for molecular surveillance of invasive pneumococcal disease (IPD). Catalan Hospitals are invited, not forced, to send invasive pneumococcal strains. For the present study, neither ethics approval nor informed consent to participate were requested as it is a proof of concept study in which samples were duly anonymized.

### Microbiological methods

An evaluation panel including a total of 32 invasive pneumococcal isolates recovered from sterile fluid samples (blood, cerebrospinal fluid and pleural fluid) was performed. Samples were obtained from pure cultures around 10^8^ UCF/ml corresponding to 0.5 McFarland in order to obtain 400 ng, the required input DNA for sequencing. All 32 strains were stored, in 1.5 mL microcentrifuge tubes with screw cap, with skimmed milk as a growth media preserved and conserved in the biobank facilities of the University Hospital Sant Joan de Deu. These strains had previously been identified and characterized by the Molecular Microbiology Laboratory with conventional methods (Quellung reaction, Multiplex PCR and Sanger-MLST) before being analysed by MinION.

### Conventional methods

#### Microbiological identification

All pneumococcal invasive isolates were cultured on blood agar plates (Columbia agar supplemented with 5% sheep blood; bioMérieux) and were identified by standard microbiological methods including optochin sensitivity test and an antigenic test.

#### Serotype analysis

Identification of capsular pneumococcal serotypes was performed using multiplex PCR combined with fragment analysis and automated fluorescent capillary electrophoresis. This technique has been used since 2010 and allows the detection of 40 serotypes/serogroups: (1, 2, 3, 4, 5, 6A/6B, 6C, 7F/7A, 7C/(7B/40), 8, 9 V/9A, 9 N/9 L, 10A, 10F/(10C/33C), 11A/11D/11F, 12F/(12A/44/46), 13, 14, 15A/15F, 15B/15C, 16F, 17F, 18/(18A/18B/18C/18F), 19A, 19F, 20, 21, 22F/22A, 23A, 23B, 23F, 24/(24A/24B/24F), 31, 33F/(33A/37), 34, 35A/(35C/42), 35B, 35F/47F, 38/25F and 39) [16]. All strains were also sent to the National Pneumococcus Reference Center of Majadahonda, Madrid, Spain, to complete serotyping by Quellung reaction and determine antimicrobial susceptibility study.

#### Clonal analysis

ST analysis was performed with MLST. MLST involves PCR amplification of seven housekeeping genes followed by Sanger DNA sequencing [17] [6]. Allele assignation and ST were carried out using the software at the pneumococcal web page http://pubmlst.org/spneumoniae/. STs are grouped into clonal complexes (CCs) by their similarity to a central allelic profile [18]. STs that shared 6 out of 7 allelic variants (SLV, single locus variants) and 5 out of 7 allelic variants (DLV, double locus variants) were considered a CC.

### MinION pipeline

#### Sample preparation

Strains were collected from our laboratory biobank. They were cultured overnight and harvested the following day.

#### DNA extraction

DNA was extracted according to manufacturer instructions of ZymoBIOMICS DNA Microprep kit (ZYMO RESEARCH) with minor changes according to the instructions of the manufacturer. Extraction procedure required about 2 h’ time per 12 samples. This is a column-based system for total nucleic acid extraction. Quantification of double strand DNA (dsDNA) through a fluorimetric assay was performed using Qubit (Life Technologies, Carlsbad, California, US) and the High Sensitivity (HS) dsDNA Assay Kit under manufacturer’s conditions. Purity was assessed through NanoDrop™ Spectrophotometer (Thermo Fisher Scientific, Waltham Massachusetts, US).

#### Library preparation and sequencing

Library preparation was performed using the Rapid Barcoding Kit (SQK-RBK004) provided by Oxford Nanopore Technologies with an R9 flow cell (FLO-MIN106). The procedure took approximately 1 h per 12 samples. MinION libraries were created with 400 ng of total genomic DNA for each sample with an adjusted volume of 7.5 μL Nuclease-free water. A clean-up step using Agencourt AMPure XP beads (Beckman coulter, Munich, Germany) at 1X concentration was used to discard short fragments as recommended by the manufacturer. DNA quantity was assessed using Qubit fluorimeter. Promega calculator (https://www.promega.es/resources/tools/biomath/) was used to convert μg dsDNA into pmol. This parameter must fall between 0.2–0.25 pmol, as specified in the protocol kit. This amount refers to the prepared library that must be loaded into the MinION flow cell. Once the library has been loaded, we initiated a standard 18 h sequencing procedure using the MinKNOW software, and by using the live basecalling feature.

#### Data analysis

During sequencing or once MinION finishes running, the data set was analysed by the real-time workflow provided by Nanopore EPI2ME platform “What’s in my pot?” (WIMP), which assigns taxonomy by comparing read sequences against a database. After sequencing, fast5 sequences were converted into FASTQ by using Albacore software. Reads were assembled using CANU for clonal analysis [19]. Once assembled, allelic variants were obtained through www.genomicepidemiology.org website. Finally, ST analysis was performed using the software at the pneumococcal web page http://pubmlst.org/spneumoniae/.

For MLST assignment from whole genome data (wgMLST), contigs generated by CANU were uploaded on PathogenWatch online tool (https://pathogen.watch/), a global platform for genomic surveillance. Conventional MLST by Sanger sequencing, which is basically only focus on 7 housekeeping genes, can be extended to wgMLST by which many more loci are considered.

#### Raw data

No administrative permissions were required to access the raw data as no human DNA was analyzed. Only bacterial DNA from *S.pneumoniae* strains was analyzed.

## Supplementary Information

**Additional file 1 Supplementary Table.** Statistics description of raw read sequences obtained by MinION.

## Data Availability

Raw metagenomic reads were submitted to NCBI Sequence Read Archive (SRA), under the bioproject number PRJNA643905.

## Abbreviations

*S.pneumoniae*: *Streptococcus pneumoniae*
MLST: Multi Locus Sequence Typing
ST: Sequence Type
WGS: Whole Genome Sequencing
IPD: Invasive Pneumococcal Disease
MIC: Minimum Inhibitory Concentrations
EUCAST: European Committee on Antimicrobial Susceptibility Testing
CC: Clonal Complex
SLV: Single Locus Variant
DLV: Double Locus Variant
dsDNA: Double strand DNA
HS: High Sensitivity
WIMP: What’s In My Pot
QLV: Quadruple Locus Variant
