# Supplementary material for: Rapid identification, capsular typing and molecular characterization of Streptococcus pneumoniae by using whole genome nanopore sequencing
Source: BMC Microbiol. 2020 Nov 13;20:347. doi: 10.1186/s12866-020-02032-x (PMC7666501; doi:10.1186/s12866-020-02032-x)
Supplement: Supplementary file 1 — Additional file 1 Supplementary Table. Statistics description of raw read sequences obtained by MinION. [file 12866_2020_2032_MOESM1_ESM.pdf]

Supplementary table. Statistics description of raw read sequences obtained by MinION.

ID	Number of reads	Mean lenght (bp)	Mean qscore	Number of contigs	Maximum lenght (bp)	Coverage	wg-MLST identity (%)
3762	3024	1298	13.17	7	2111595	1052.36	99.01
3201	30930	5423	10.50	1	2128806	1768.90	99.25
3490	20679	4820	10.51	2	2159668	3948.76	99.09
3581	34944	4722	10.49	1	2202870	2664.30	99.25
3603	30374	3805	10.47	2	2057021	3941.13	99.17
3612	34412	3045	10.43	2	2159955	7435.61	99.01
3618	50087	3526	10.47	1	2165118	256.12	99.17
3621	33255	4250	10.52	3	2197221	3584.07	98.27
3626	17650	2337	10.41	7	683933	1562.47	98.51
3630	20949	3704	10.43	1	2069070	6411.62	98.97
3631	26972	3524	10.45	2	2232160	8030.20	99.01
3961	35432	3365	10.46	2	2145085	6480.00	99.17
3814	46773	2704	10.44	3	2088025	6248.94	99.17
2911	27258	5212	10.52	1	2043579	2215.31	98.76
3055	16226	7706	10.57	2	2047835	1998.96	98.76
3058	20406	4901	10.53	4	2099078	348.80	98.76
3081	15004	4905	10.52	4	2151932	3055.51	98.14
3082	9521	5926	10.51	1	2138875	2181.76	98.97
3460	19653	7535	10.59	1	2021009	1197.78	98.76
3749	29289	5020	11.03	6	2200961	3811.79	98.76
3547	41818	4620	11.02	2	2130862	1884.41	99.13
3964	20946	5665	11.18	5	2139833	3390.37	99.50
3878	8019	5267	11.14	4	2010632	1503.36	98.76
3011	20023	5478	10.76	1	2202755	3541.48	98.55
3012	11616	4019	10.69	5	2241267	3620.58	99.01
3052	20546	5730	10.76	2	2115730	2373.46	99.17
3077	11839	4399	10.75	3	2135344	2664.97	97.94
3243	37101	5293	10.79	4	2161861	1606.17	99.17
3418	35979	7276	10.82	1	2019879	3922	99.01
3509	15765	4038	10.76	3	2114490	3019.99	99.17
3690	11108	3388	10.72	4	1738962	1735.17	98.51
3791	14522	7283	10.76	6	2162386	2952.96	98.55
